# Supplementary material for: Comment on 'AIRE-deficient patients harbor unique high-affinity disease-ameliorating autoantibodies'
Source: eLife. 2019 Jun 27;8:e43578. doi: 10.7554/eLife.43578 (PMC6597240; doi:10.7554/eLife.43578)
Supplement: Transparent reporting form [file elife-43578-transrepform.pdf]

eLIFE
1st Floor
24 Hills Road
Cambridge CB2 1JP, UK
P 01223 855340
W elifesciences.org
T @elife

eLife's transparent reporting form

We encourage authors to provide detailed information *within their submission* to facilitate the interpretation and replication of experiments. Authors can upload supporting documentation to indicate the use of appropriate reporting guidelines for health-related research (see EQUATOR Network), life science research (see the BioSharing Information Resource), or the ARRIVE guidelines for reporting work involving animal research. Where applicable, authors should refer to any relevant reporting standards documents in this form.

If you have any questions, please consult our Journal Policies and/or contact us: editorial@elifesciences.org.

Sample-size estimation

- You should state whether an appropriate sample size was computed when the study was being designed
- You should state the statistical method of sample size computation and any required assumptions
- If no explicit power analysis was used, you should describe how you decided what sample (replicate) size (number) to use

Please outline where this information can be found within the submission (e.g., sections or figure legends), or explain why this information doesn't apply to your submission:

In the present study we used previously reported protein microarray data (Landegren et al. Sci Transl Med 2015 and Scientific Reports 2015). Power analysis was not used to decide the number of samples to investigate in the original study. We divided our full cohort of 93 patients with autoimmune polyendocrine syndrome type 1 (APS1) into a discovery cohort of 51 patients, which were subjected to protein array screening, and a validation cohort of 42 patients, where identified candidate autoantigens were confirmed using independent methods. 21 healthy controls were included in the protein array screening experiment. Several hundred healthy and disease control subjects were included in the validation phase.

Replicates

- You should report how often each experiment was performed
- You should include a definition of biological versus technical replication
- The data obtained should be provided and sufficient information should be provided to indicate the number of independent biological and/or technical replicates
- If you encountered any outliers, you should describe how these were handled
- Criteria for exclusion/inclusion of data should be clearly stated
- High-throughput sequence data should be uploaded before submission, with a private link for reviewers provided (these are available from both GEO and ArrayExpress)

Please outline where this information can be found within the submission (e.g., sections or figure legends), or explain why this information doesn't apply to your submission:

eLife Sciences Publications, Ltd is a limited liability non-profit non-stock corporation incorporated in the State of Delaware, USA, with company number 5030732, and is registered in the UK with company number FC030576 and branch number BR015634 at the address 1st Floor, 24 Hills Road, Cambridge CB2 1JP | August 2014

Previously reported protein microarray data (Landegren et al. Sci Transl Med 2015 and Scientific Reports 2015) was used. Suspected printing contamination artifacts were identified and excluded, as previously described (Landegren et al. Scientific Reports 2015). No other data was excluded. The protein array screening experiment and data analyses are described in the methods section. Further information is available in our previously published articles (Landegren et al. Sci Transl Med 2015 and Scientific Reports 2015), which are referenced in the manuscript .

Statistical reporting

- Statistical analysis methods should be described and justified
- Raw data should be presented in figures whenever informative to do so (typically when N per group is less than 10)
- For each experiment, you should identify the statistical tests used, exact values of N, definitions of center, methods of multiple test correction, and dispersion and precision measures (e.g., mean, median, SD, SEM, confidence intervals; and, for the major substantive results, a measure of effect size (e.g., Pearson's r, Cohen's d)
- Report exact p-values wherever possible alongside the summary statistics and 95% confidence intervals. These should be reported for all key questions and not only when the p-value is less than 0.05.

Please outline where this information can be found within the submission (e.g., sections or figure legends), or explain why this information doesn't apply to your submission:

The statistical analyses that were used are described in the methods section under the heading "Data analysis".

(For large datasets, or papers with a very large number of statistical tests, you may upload a single table file with tests, Ns, etc., with reference to sections in the manuscript.)

Group allocation

- Indicate how samples were allocated into experimental groups (in the case of clinical studies, please specify allocation to treatment method); if randomization was used, please also state if restricted randomization was applied
- Indicate if masking was used during group allocation, data collection and/or data analysis

Please outline where this information can be found within the submission (e.g., sections or figure legends), or explain why this information doesn't apply to your submission:

Patients with APS1 were studied as cases and healthy blood donors served as controls in our investigation of the autoantigen repertoire in APS1.<br><br>In the study of neutralizing interferon autoantibodies in APS1, three groups were assessed: APS1 patients diagnosed with type 1 diabetes, APS1 patients without type 1 diabetes, and healthy controls. Serum from a patient with IFNγ autoantibodies and disseminated mycobacterial infection was also included as a control.<br><br>Information regarding group allocation was not blinded during the experiments or data analysis.

Additional data files ("source data")

- We encourage you to upload relevant additional data files, such as numerical data that are represented as a graph in a figure, or as a summary table
- Where provided, these should be in the most useful format, and they can be uploaded as "Source data" files linked to a main figure or table
- Include model definition files including the full list of parameters used
- Include code used for data analysis (e.g., R, MatLab)
- Avoid stating that data files are "available upon request"

Please indicate the figures or tables for which source data files have been provided:

In this work we used protein array data that was generated in a previous study. Results from this dataset has been included in two previous publications (Landegren et al Sci Transl Med 2015 and Scientific Reports 2015). We are positive to an exchange of protein array datasets with Meyer et al. and to upload the data to a public database.
